# Response to “Misunderstanding the Match: Do Students Create Rank Lists Based on True Preferences?”

**DOI:** 10.5811/westjem.2021.4.52801

**Published:** 2021-07-20

**Authors:** Benjamin H. Schnapp, Katie Ulrich, Jamie Hess, Aaron S. Kraut, David Tillman, Mary Westergaard

**Affiliations:** University of Wisconsin School of Medicine and Public Health, BerbeeWalsh Department of Emergency Medicine, Madison, Wisconsin

We agree fully that “reciprocal liking” may be an important causal factor behind some of the mismatch between student behavior and theoretically ideal Match behavior. Indeed, it likely explains why programs and applicants go out of their way to communicate liking for one another despite official National Resident Matching Program (NRMP) policy discouraging communications.^1^ It is well supported in the social psychology literature that expressing liking for someone increases the tendency for the other individual to like them.^2^–^4^ Additionally, we agree that programs often have a good sense of where they can provide maximal value to applicants for career development, such as mentorship, research infrastructure, or specific clinical experiences such as flight medicine. We would advise applicants against making more than minor changes to their rank lists based on communications from programs regarding these factors, but we agree that it is not necessarily irrational for an applicant to adjust their rank list when a program communicates strong interest.

However, there are several reasons to believe that the findings of this study are not comprehensively explained by students making potentially justifiable adjustments to their rank list. First, when asked directly if perceived competitiveness would impact their rank list, 63% of students responded that it would by at least a moderate amount, suggesting that it is not a sense of liking or a strong value proposition that is causing students to make changes to their list.

Second, to attempt to account for the effect of potential “reciprocal liking,” we created one of our case scenarios to depersonalize the rank decision. Specifically, the scenario stated that the applicant was being ranked lower because of a decision to prioritize internal applicants, removing any potential judgment of the applicant by the program. Despite not being “disliked” by the program in this scenario, 22% of respondents still stated that they would move the program lower on their rank list, while 3% would move it higher. We believe this scenario is particularly relevant, as programs may place applicants lower on their rank list for a variety of reasons beyond perceived potential for success, including a desire to create a residency class with diverse backgrounds, interests and aspirations. Sound “reciprocal liking” and “fit”-based decision making also do not explain why students did not change their rank lists when the facts of the scenario suggested that they should have (e.g., a partner’s amazing job offer).

Third, we would strongly caution both programs and applicants against over-reliance on a subjective assessment of “fit” to override their otherwise methodologically sound rankings. While “fit” is known to be used heavily by applicants and programs alike, it is also a known proxy for similarity to the status quo and can bias programs and applicants against otherwise strong matches that may enable them to grow and change in unexpected ways.^5^

Some of the nuances of why students do not display consistently logical behavior when making rank lists still remain to be elucidated. It remains possible that subjects misinterpreted the case scenarios, for example. We feel that our overall findings, however, are still most consistent with some level of student misunderstanding of the Match algorithm. We believe that our original recommendation for more specific education for senior students about how the Match functions is well-founded based on the results of this study.
